# New Promising Therapeutic Avenues of Curcumin in Brain Diseases

**DOI:** 10.3390/molecules27010236

**Published:** 2021-12-31

**Authors:** Tarek Benameur, Giulia Giacomucci, Maria Antonietta Panaro, Melania Ruggiero, Teresa Trotta, Vincenzo Monda, Ilaria Pizzolorusso, Dario Domenico Lofrumento, Chiara Porro, Giovanni Messina

**Affiliations:** 1 Department of Biomedical Sciences, College of Medicine, King Faisal University, Al-Ahsa 31982, Saudi Arabia; tbenameur@kfu.edu.sa; 2 Department of Neuroscience, Psychology, Drug Research and Child Health, University of Florence, 50134 Florence, Italy; giuliagiacomucci.md@gmail.com; 3 Biotechnologies and Biopharmaceutics, Department of Biosciences, University of Bari, 70125 Bari, Italy; mariaantonietta.panaro@uniba.it (M.A.P.); melania.ruggiero@uniba.it (M.R.); 4 Department of Clinical and Experimental Medicine, University of Foggia, 71121 Foggia, Italy; teresa.trotta@unifg.it (T.T.); vincenzo.monda@unicampania.it (V.M.); Giovanni.messina@unifg.it (G.M.); 5 Unit of Dietetic and Sport Medicine, Section of Human Physiology, Department of Experimental Medicine, Luigi Vanvitelli University of Campania, 81100 Naples, Italy; 6 Child and Adolescent Neuropsychiatry Unit, Department of Mental Health, ASL Foggia, 71121 Foggia, Italy; ilaria.pizzolorusso@virgilio.it; 7 Department of Biological and Environmental Sciences and Technologies, Section of Human Anatomy, University of Salento, 73100 Lecce, Italy; dario.lofrumento@unisalento.it

**Keywords:** curcumin, natural flavonoid, neuroinflammation, anti-inflammatory, neurodegenerative diseases, Alzheimer’s diseases, Parkinson’s diseases, multiple sclerosis, glioblastoma multiforme, epilepsy

## Abstract

Curcumin, the dietary polyphenol isolated from *Curcuma longa* (turmeric), is commonly used as an herb and spice worldwide. Because of its bio-pharmacological effects curcumin is also called “spice of life”, in fact it is recognized that curcumin possesses important proprieties such as anti-oxidant, anti-inflammatory, anti-microbial, antiproliferative, anti-tumoral, and anti-aging. Neurodegenerative diseases such as Alzheimer’s Diseases, Parkinson’s Diseases, and Multiple Sclerosis are a group of diseases characterized by a progressive loss of brain structure and function due to neuronal death; at present there is no effective treatment to cure these diseases. The protective effect of curcumin against some neurodegenerative diseases has been proven by in vivo and in vitro studies. The current review highlights the latest findings on the neuroprotective effects of curcumin, its bioavailability, its mechanism of action and its possible application for the prevention or treatment of neurodegenerative disorders.

## 1. Introduction

Recent evidence suggests that the use of nutraceuticals, dietary supplements may bring protection to central nervous system (CNS) by preserving neurons against stress-induced damage, by suppressing neuroinflammation and by increasing the neurocognitive performance.

Curcumin is one of the curcuminoid constituents present in turmeric (*Curcuma longa Linn*) and is a perennial herb of the Zingiberaceae family. Turmeric, also called “golden spice” is used as remedy in traditional medicine and is also widely used in the Asian cuisine as a food additive and as a coloring agent in the beverage industry [1].

The (1E,6E)-1,7-bis(4-hydroxy-3-methoxyphenyl)-1,6-heptadiene-3,5-dione is the IUPAC name of curcumin, its chemical formula is C_21_H_20_O_6_ and it has a molecular weight of 368.38 g/mol.

Various biological activities and therapeutic properties of curcumin are due to its chemistry, in particular phenolic hydroxyl groups, the central bis-α, β-unsaturated β-diketone, double-conjugated bonds, and methoxy groups are responsible for its bio-pharmacological effects.

Curcumin is a lipophilic molecule, with poor solubility in water or hydrophilic solutions, instead it is easily soluble in organic solvents such as methanol, ethanol, acetone and dimethyl sulfoxide, chloroform [2].

Curcuminoid complex contains curcumin, demethoxycurcumin and bis-demethoxycurcumin [3].

Curcumin, like other phytochemicals, has pleiotropic activity on cells, in fact due to his ability to interact with many proteins, curcumin can provoke cellular responses to external stimuli. In addition, curcumin up- and down-regulates various miRNA and can cause epigenetic changes in cell.

Several in vitro, in vivo and clinical trials have focused on the potential therapeutic effects of curcumin including antioxidant [4], immunomodulatory, cardio-protective [5], nephro-protective [6], hepato-protective [7,8], anti-neoplastic [9,10], anti-microbial, anti-diabetic [11], anti-rheumatic [12] anti-aging [13], anti-inflammatory especially anti-neuroinflammatory [14], as well as inhibiting properties for microglia [15].

Despite its numerous therapeutic benefits, this bioactive compound has poor bioavailability due to insufficient absorption, chemical instability, and rapid metabolism in the body.

In order to increase the bioavailability of curcumin, nanocarriers have been proved to be a promising strategy, to enhance its therapeutic effects.

Due to their nanometric size and chemical property, nanoparticles [16], liposomes [17,18], micelles, phospholipid vesicles [19] and polymeric nanoparticles [20,21] are able to increase the effectiveness of curcumin.

Among the natural nanocarriers, extracellular vesicles, especially exosomes, are used as a system for drug delivery. Exosomes are released from cells by exocytosis after the maturation of multivesicular bodies.

Exosomes are able to mediate cellular communication with their protein, lipid and nucleic acid composition [22]. The lipid membrane of the exosome contains curcumin by the interaction between the hydrophobic tails and hydrophobic active ingredient. The insertion in the lipid bilayer guarantees the protection of curcumin from degradation [23]. In fact, curcumin with an exosomal formulation is more effective respect to liposomal curcumin and free curcumin [23].

Zhang et al. have demonstrated that intranasal administrated curcumin-loaded exosomes in inflammation-mediated disease models, such as Lipopolysaccharide (LPS) -induced brain inflammation model, experimental autoimmune encephalitis, and a GL26 brain tumour model, induce neuroprotection by reducing neuroinflammation or tumour size [24].

In ischemia-reperfusion (I/R) injuries, curcumin-loaded exosomes are able to down-regulate reactive oxygen species (ROS) production in lesions, reduce blood–brain barrier (BBB) damage and suppress mitochondria-mediated neuronal apoptosis [25].

Liposomes are nanovesicles made up of single or multiple bilayers of phospholipids that enclose hydrophilic, lipophilic, and amphiphilic molecules [26], that could be used to deliver drugs into target sites.

Mohajeri et al. have demonstrated the anti-inflammatory and anti-oxidant effects of polymerized nano-curcumin which had positive effects on an experimental autoimmune encephalomyelitis model of multiple sclerosis, and induced myelin repair mechanisms [27].

Nano-curcumin has neuroprotective effects on early brain injuries, it is in fact able to attenuate BBB dysfunction following subarachnoid hemorrhage by preventing the destruction of the tight junction protein (ZO-1, occludin, and claudin-5). In addition, nano-curcumin up-regulates the glutamate transporter-1 which reduces the glutamate concentration in cerebro spinal fluid (CSF) following subarachnoid hemorrhage and inhibits the activation microglia [28].

A combination of ω*-3* fatty acids and nano-curcumin significantly reduces the frequency of migraine attacks by modulation of IL-6 gene expression and C-Reactive Protein levels, as evidenced in a set of clinical trial [29]. CUR-loaded liposomes reduce angiotensin-converting enzyme activity in target regions of the brain and potentiate memory restoration in rats with Alzheimer’s disease (AD) [30].

As life expectancy increases worldwide, neurodegenerative diseases increase and this leads to a greater burden of socio-economic discomfort for patients, families and communities [31]. Neurodegenerative diseases are characterized by disorders that conduct to a progressive disruption of the structure and/or function of neurons and of their synaptic network that finally induces a loss of brain function.

AD, Parkinson’s disease (PD), Huntington’s disease (HD), Multiple Sclerosis (MS), and amyotrophic lateral sclerosis (ALS) are the most common neurodegenerative diseases present in the elderly.

Factors that lead to neurodegenerative diseases include genetic polymorphisms, increasing age, gender, poor education, endocrine diseases, oxidative stress, inflammation, stroke, hypertension, diabetes, smoking, head trauma, depression, infection, tumors, vitamin deficiencies, immune and metabolic disorders, and chemical exposure [32].

The inflammatory response within the brain or spinal cord is known as neuroinflammation.

Neuroinflammation is common in a number of brain diseases, including AD, PD, MS and many others. This process is mediated through the production of cytokines, chemokines, reactive oxygen species, and secondary messengers, which could destroy the BBB, resulting in cell damage and loss of neuronal functions [33]. Glia, endothelial cells, and peripherally derived immune cells produced these mediators.

Among the glial cells, microglia and astrocytes play a central role in the pathophysiology of neurodegenerative diseases. Astrocytes work together to maintain CNS homeostasis and promote neuronal survival by regulating metabolite traffic and blood flow. Microglial cells perceive the disturbance of brain tissue homeostasis, and function as CNS phagocytes [34,35]. The purpose of this review is to emphasize the importance of curcumin in the treatment of AD, PD, MS glioblastoma and epilepsy focusing on its potential mechanism of action in improving their course.

## 2. Curcumin and AD

AD represents the main cause of dementia worldwide, accounting for 60–80% of cases who are diagnosed with dementia [36]. Clinically, AD is typically featured by memory loss, progressive cognitive decline and impairment of previous levels of functioning and performing at work or at usual activities. Neurodegeneration has been attributed and is driven by extracellular aggregates of amyloid β (Aβ) plaques and intracellular neurofibrillary tangles (NFTs) made of hyperphosphorylated tau protein in cortical and limbic areas of the human brain [37]. The formation of Aβ plaques starts from the anomalous processing of amyloid precursor protein (APP) by β-secretases (BACE1) and γ-secretases, leading to the production of different types of Aβ monomers, among which Aβ40 and Aβ42 (highly insoluble and aggregation-prone). As a result, Aβ monomers continue to oligomerize and aggregate into plaques. NFTs are the second pathological hallmark of AD and consist of hyperphosphorylated tau localized in cytoplasm of neurons [38]. Tau has a microtubule-binding domain and assembles with tubulin, resulting in the formation of stable microtubules. Aβ may activate several kinases, including glycogen synthase kinase 3 β (GSK-3β), cyclin-dependent kinase 5 (CDK5), and others like Protein Kinase C, Protein Kinase A, extracellular signal-regulated kinase 2 (ERK2), a serine/threonine kinase, which phosphorylate tau, leading to its oligomerization [39]. As a consequence, microtubules become unstable, and their subunits transform into big chunks of tau filaments, which further aggregate into NFTs. NFTs are highly insoluble and lead to an abnormal loss of communication between neurons and signal processing and finally apoptosis in neurons [40]. According to the amyloid hypothesis, pathological alterations of tau are considered to be downstream events of Aβ deposition. However, it has also been hypothesized that Aβ and tau act in parallel pathways that cause AD and amplify each other’s toxic effects [41].

Given the social and economic impact, it is important to understand which risk factors could influence the development of AD and also to find medications that can prevent the onset or stop the disease course. At the state of art, there are limited number of drugs that are available for the treatment of AD, such as acetylcholinesterase inhibitors (donepezil, rivastigmine and galantamine) and glutamate antagonist memantine, which are not effective in stopping the disease progressive course [42]. Recently, the FDA approved the use of the first drug with a putative disease-modifying mechanism, Aducanumab, which is a human monoclonal antibody that selectively reacts with Aβ aggregates and reduces Aβ plaques in the brain, thus predicting important clinical benefits. However, post-approval clinical trials are needed to verify the real drug’s clinical benefit [43]. Several natural compounds have been recently investigated to better understand their potential efficacy in the “treatment” of AD [44]. Current research is focused on curcumin’s mechanism of action and its role in modulation of AD progression.

**Figure 1:**
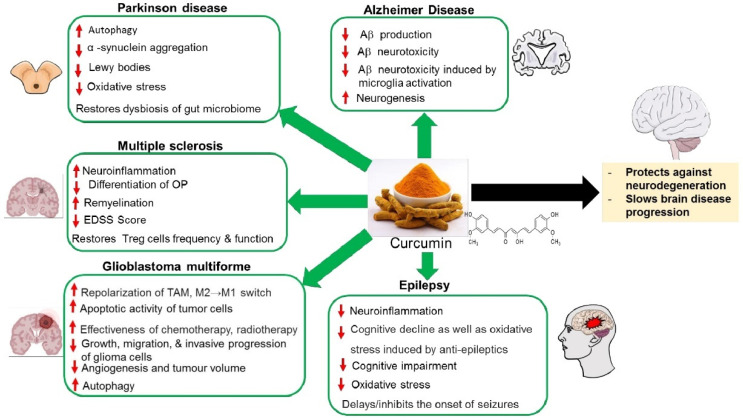
The neuroprotective effects of curcumin against neurological disorders and associated symptoms. TAM: tumor-associated macrophages; OP: Oligodendrocye progenitor; EDSS: The Expand Disability Status Scale.

Curcumin’s mechanisms of action are pleiotropic (Table S1) [45] and target both Aβ and tau (see Figure 1). Moreover, it modulates other aspects of the disease process: it also binds copper, lowers cholesterol levels, modifies microglial activity, inhibits acetylcholinesterase, enhances the insulin-signaling pathway, and acts as an antioxidant [45]. Curcumin seems to target Aβ at different levels. In fact, it has been described that it inhibits Aβ production; moreover, curcumin inhibits the aggregation both in vitro and in mouse models thus preventing the formation of plaques and it promotes disaggregation of fibrillar form [46].

Concerning Aβ production, in vitro studies showed that curcumin acts as an inhibitor of BACE1, which is involved in the cleavage of APP [47]. These results were confirmed in mouse models of AD, demonstrating that curcumin downregulates the expression of BACE1, thus reducing Aβ formation [48].

**Figure 2:**
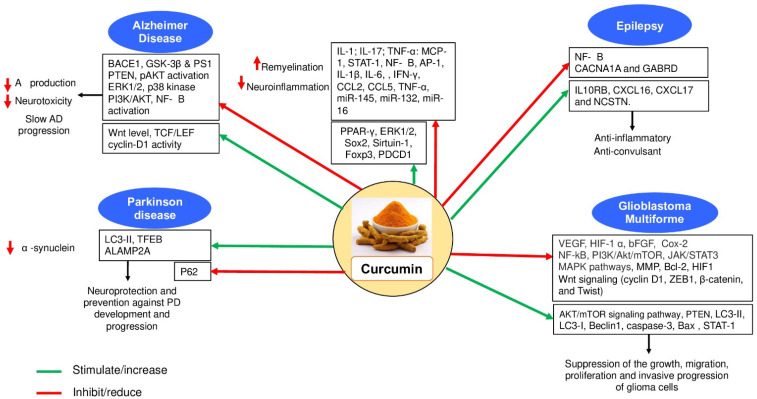
Molecular mechanisms by which curcumin exerts its therapeutic effects on neurological disorders.

In addition, curcumin appears to inhibit the GSK-3β-dependent presenilin 1 (PS1) activation and consequently to reduce Aβ production. In fact, neuroblastoma SHSY5Y cells treated with curcumin showed a marked decreasing of PS1 and GSK-3β levels and a marked reduction of Aβ production in a dose- and time- dependent manner [49]. GSK-3β is activated when it is dephosphorylated at Ser9 site. Its activity is regulated upstream by Akt, a serine/threonine-specific protein kinase. Phosphatidylinositol (PIP) and PDK mediated phosphorylation of Akt at Ser473 and Thr308 sites leads to Akt activation and consequent phosphorylation and inhibition of GSK-3β. Akt activity is negatively regulated by PTEN, which catalyzes phosphoinositide to dephosphorylate deactivating PIP3 signaling. PI3K/Akt/GSK-3β signaling pathway is also directly affected by Aβ exposure [50], indeed, oligomers active GSK-3β through dephosphorylation at Ser9 site. Moreover, Aβ induces a downregulation of the phosphorylation of Akt and also an overexpression of PTEN, its negative regulator, which leads to a downstream activation of GSK-3β. Curcumin inhibits both overexpression of PTEN mRNA, and the downregulation of phosphorylation-mediated activation of Akt, and also Aβ-mediated GSK-3β activation [51,52], thus reduced Aβ production and build-up of plaques (Figure 2).

Regarding the role of curcumin in inhibiting the aggregation of Aβ, it has been suggested that curcumin destabilizes the attractive forces required for the formation of β-sheets in amyloid plaques through its hydrophobicity or its interaction between the keto or enol rings and aromatic ring of Aβ dimers [53]. The destabilization of β-sheets is also influenced by the interaction between curcumin’s hydroxyl groups on the aromatic rings and the polar pockets of Aβ [54].

Interestingly, recent in vitro studies have focused on curcumin’s role in preventing Aβ neurotoxicity. Thapa et al. showed that curcumin reduces the rate of Aβ insertion into the plasma membrane and consequently acts as a protective factor against Aβ membrane toxicity. In more details, curcumin reduced the disruption of plasmatic membrane due to Aβ, thus avoiding elevated calcium influx and cell death [55]. The neuroprotective effect of curcumin, probably membrane-mediated, seems to act by reducing toxicity induced by a wide range of Aβ conformers, including monomeric, oligomeric, pre-fibrillary and fibrillary Aβ [56]. Interestingly, it has also been described that curcumin promotes the formation of “off-pathway” soluble oligomers and pre-fibrillar aggregates that are non-toxic [56]. Another study by Huang et al. showed that curcumin is able to attenuate Aβ-mediated activation of the NMDA receptor of glutamate and thus inhibits the intracellular increase in Ca^2+^, which is involved in glutamate toxicity. The effect of curcumin on the depression of the NMDA receptor/Ca^2+^ pathway seems to prevent cell damage induced by Aβ [57]. Despite these interesting results, in vivo studies are yet necessary to translate these findings and find a potential clinical use.

Concerning NFTs, GSK-3β regulates the phosphorylation of tau by adding phosphate groups on serine and threonine amino acid residues. Curcumin has been shown to prevent the hyperphosphorylation of tau acting as a GSK-3β inhibitor [45,47]. In more details, Huang et al. [51] showed that curcumin inhibits Aβ-induced tau hyperphosphorylation involving PTEN/Akt/GSK-3β pathway in human cell cultures and consequently influences the inhibition of tau hyperphosphorylation preventing aggregation in NFTs.

Curcumin may also play a role in NFTs clearance with a consequent reduction in tau-induced toxicity. Indeed, in mouse neuron cell cultures, curcumin, at low concentration, upregulates the expression of BCL2 associated athanogene 2 (BAG2), a molecular chaperone that delivers tau to the proteasome for degradation [58]. However, since this study was not performed on pathological neurons, these results need to be confirmed. Another study by Miyasaka et al. described those levels of acetylated α-tubulin, an indicator of microtubule stabilization, was significantly greater in curcumin-treated nematodes, suggesting that curcumin may mitigate tau-mediated neurotoxicity by improving microtubule stabilization [59].

Besides Aβ and NFTs, other factors should be taken into account in AD pathogenesis. Microglia have a critical role in the innate immune response of the CNS and can be classified in M1 (which secretes neurotoxic cytokines, prostaglandins, ROS and nitric oxide) and M2 phenotype (which releases neuroprotective and anti-inflammatory mediators and phagocyte toxic protein aggregates). The role of microglia in AD has been deeply studied [60]. Aβ deviates microglia from neuroprotective M2 to neurotoxic M1 phenotype [61]. Additionally, Aβ accumulation activates microglia, which produces inflammatory mediators thus promoting further Aβ accumulation, leading to this positive feedback loop. Curcumin appears to play a role in reducing neurotoxicity due to Aβ induced microglia activation [62]. In this regard, it was reported that curcumin blocks ERK1/2 and p38 kinase signaling in Aβ activated microglia thus reducing the production of TNF-α, IL-1β and IL-6 [63] and, in addition, attenuates the release of nitric oxide [64]. Moreover, curcumin suppresses phosphoinositide 2 kinase (PI3K)/Akt phosphorylation and the activation of nuclear factor κB (NF-κB), which drive microglia activation and neuroinflammation pathways [64]. Interestingly, curcumin induces the increasing of the peroxisome proliferator-activated receptor γ (PPARγ) protein levels, thus enhancing PPARγ anti-inflammatory activity in downregulation of NF-κB and ERK pathways. On the other hand, curcumin may enhance the neuroprotective effect of M2 microglia: in fact, Aβ phagocytosis seems to be increased in microglia from AD patients treated with curcuminoids in vitro [65].

A significant reduction in neurogenesis has been widely described in AD and other neurodegenerative diseases [66]. Previous works found that curcumin regulates neurogenesis through the activation of Wnt pathway in vitro and in the hippocampus and subventricular zone of adult rats. Wnt interacts with the 7-transmembrane Frizzled receptor and phosphorylated co-receptor low-density lipoprotein (LRP-5/6), thus leading to the activation of cytoplasmic disheveled (Dvl) protein. Once activated, Dvl protein interacts with Axin/APC/GSK-3β destruction complex and inhibits GSK-3β. The inhibition of GSK-3β leads to accumulation of cytoplasmic β-catenin and its translocation into the cell nucleus. In the nucleus, β-catenin interacts with the TCF/LEF promoter complex, leading to activation of target genes that are involved in proliferation and differentiation of CNS. Curcumin seems to influence this pathway at different levels. In more details, curcumin interacts with Wif-1 and Dkk-1, which are Wnt inhibitory molecules, thus increasing Wnt levels. Moreover, curcumin may likely interact with GSK-3β, thus enhancing the levels of cytoplasmic β-catenin, and enhances β-catenin nuclear translocation, leading to enhanced TCF/LEF and cyclin-D1 promoter activity and increased neurogenesis. Interestingly, it has been showed that although low brain concentrations of curcumin (500 nM) stimulated neurogenesis, high brain concentrations (10 μM) inhibited neurogenesis and neuroplasticity [67]. Therefore, the choice of concentration of curcumin should be carefully chosen. Preclinical models have predominately demonstrated a positive effect of curcumin on AD, however, only a limited number of clinical studies has examined curcumin’s effect on human cognitive functioning in AD and results are less consistent. The findings on Aβ reduction are ambiguous, since no significant changes in the Aβ or tau levels in plasma or CSF were found between curcumin and placebo [68,69]. On the other hand, neuroimaging supports that curcumin reduces Aβ deposits in the brain on 2-(1-{6-[(2-[F- 18]fluoroethyl)(methyl)amino]-2-naphthyl}ethylidene) malononitrile positron emission tomography (FDDNP-PET) in non-demented patients [70]. These inconsistencies may be related to differences in methodology and the included population [71]. Moreover, curcumin shows low bioavailability and its effects on antioxidant pathways and neurogenesis probably need more time to induce a significant improvement in cognitive capacity and in Aβ reduction. Thus, the mild effects previously described could also be due to the relatively short duration of treatment. Further studies are needed to improve curcumin’s bioavailability and to better explore curcumin’s effect on Aβ and NFTs, in order to understand if curcumin may be as a new potential contributor in prevention and treatment of AD.

## 3. Therapeutic Effects of Curcumin in PD

PD is the second most common neurodegenerative diseases after AD. An estimated 10 million people are suffering from PD worldwide in 2020 (https://www.epda.eu.com/, accessed on 27 October 2021) [72]. PD predominantly affects dopamine-producing neurons in substantia nigra of the midbrain leading to severe motor and cognitive dysfunction. In idiopathic PD, the pathophysiological mechanisms include the production of α-synuclein and mitochondrial respiratory dysfunction-affecting complex I, caused by ROS [73]. It is also characterized by the accumulation of protein aggregates, consisting mainly of α-synuclein, due to failure of protein degradation mechanisms such as the lysosomal system [74,75]. Most of the existing treatment modalities are only symptomatic. This includes a dopamine supplement that temporarily control the motor dysfunction caused by the degeneration of dopaminergic nigrostriatal system. Deep brain stimulation (DBS) is used in drug-resistant PD.

To prevent oxidative stress and reduce disease progression, the use of natural antioxidants remains a potential alternative therapy. Given the neuroprotective, anti-neuroinflammatory and the anti-oxidant effects against stress-induced neurodegeneration of curcumin, here we discuss the recent findings related to the beneficial effects of curcumin in reducing PD progression and prevention [12].

Although the pathogenesis of PD is still widely unclear, several mechanisms have been proposed and various evidence supports the important role of mitochondrial dysfunction in the PD pathogenesis [76].

A recent study reports the protective effects of curcumin against mitochondrial dysfunction and cell death in a siRNA-mediated PINK1 knock-down model of PD [77]. Another study describes the effects of curcumin on mitochondrial dysfunction in a paraquat-induced toxicity model of PD, in fibroblasts derived from *LRRK2*-mutation positive PD and health control. In fact, pre-treating this cell model with curcumin prior to paraquat treatment, improved maximal respiration and ATP-associated respiration without affecting the respiratory capacity. After the paraquat treatment, the post-treatment of fibroblasts with curcumin, did not improve mitochondrial respiration across the three parameters (maximal respiration, ATP-associated respiration, and spare respiratory capacity), thus suggesting the preventive effect of curcumin before the onset of PD [78].

A recent study by Motawi et al. [79] investigating the effects of curcumin and dietary supplements on the rotenone mouse model of PD showed an overall statistically significant improvement. Indeed, the administration of curcumin in of rotenone-treated mice improved α-synuclein level and reduced Lewy bodies. The behavior of animals was also improved and the levels of inflammatory mediators were significantly reduced in curcumin-treated mice when compared to the control group. These include IL-6, CRP and Ang II, previously shown with pro-inflammatory and pro-fibrotic effects that contribute to the progressive deterioration of organs function in PD [80]. When evaluating the PD markers, a significant decrease in the adenosine A2AR gene expression level was found in the mouse treated with curcumin compared to the rotenone group. Another promising improvement in the dopamine and serotonin levels was noted in curcumin-treated mouse models of PD. In addition, treatment with curcumin leads to reduced oxidative stress in PD mouse models [79]. Other supportive evidence showing similar results on rats’ models of PD with higher responses of rats to curcumin treatments regarding the oxidative stress and energetic indices. Therefore, curcumin attenuated the severe effects of PD in the rat model and can be viewed as a potential dietary supplement [81].

Evidence from the literature has shown that the impairment of the autophagy-lysosome pathway (ALP) plays a crucial role in the pathogenesis of PD. A recent study focused on the effect of curcumin on the alpha-synuclein (αS) oligomer through a molecular dynamic simulation method showed that curcumin reduced the structural stability of the α S-oligomer by perturbing its general properties. Furthermore, the aggregation of α-synuclein oligomers was prevented and the formation of fibril formation was inhibited by curcumin [82].

Because of the ability of curcumin in reducing misfolded α-synuclein by promoting autophagy, recent studies have investigated its effects on autophagy regulation. Thus, the treatment of cellular model for PD has shown an increased expression of microtubule-associated protein 1 light chain 3 (LC3-II), nuclear plasma protein determination of nuclear transcription factor EB (TFEB) and autophagy-related protein lysosome membrane protein 2 (ALAMP2A). This results in promoting autophagy-lysosome synthesis and autophagic clearance of α-synuclein [83,84].

TFEB has been identified as one of the critical key regulators of autophagy and lysosome biogenesis [85,86]. This has reinforced the hypothesis that TFEB can be considered as a new therapeutic target of PD. In fact, curcumin derivative, called E4 (curcumin analog), was able to activate and promote the translocation of TFEB from the cytoplasm into the nucleus. This translocation is accompanied by the stimulation of autophagy and lysosomal biogenesis. Mechanistically, compound E4 activated TFEB via the inhibition of AKT-MTORC1 pathway. Additionally, in the PD cell models, E4 has been shown to reduce α-synuclein levels and protect against the cytotoxicity of MPP^+^ (1-methyl-4-phenylpyridinium ion) in neural cells. These promising data showing the in vitro protective effects of E4 however still requires further in vivo experimental tests since brain bioavailability of E4 is still not known. The neuroprotective efficacy of E4 needs to be further explored in PD animal models [87].

In addition, in vivo intraperitoneal injection of curcumin promoted LC3-II protein expression and inhibited P62 expression in favor of autophagy. Curcumin inhibited α-synuclein expression and the apoptosis of dopamine neurons in the MPTP-induced PD mouse model (curcumin 80 mg/kg for 14 days) and improved the movement disorder in the mouse [33]. It has been shown that sevoflurane anesthesia induces cognitive impairment by activating autophagy in the hippocampus of the young mice [88]. Interestingly, curcumin was able to modulate autophagy at 300 mg/kg for six days and inhibit the memory impairment in mice induced by sevoflurane [89]. The protective effects of curcumin were investigated in administered orally in a 6-hydroxydopmine (6-OHDA)-induced animal model of PD. The neuroprotective effects of curcumin at (200 mg/kg) 2 weeks pre- and post-surgery were assessed by the morphological and behavioral analyses. Motor function was assessed three weeks after the surgery. Curcumin has significantly improved the abnormal motor behavior and was shown to protect against the reduced dopaminergic neurons in the substantia nigra and caudate-putamen nucleus as demonstrated by tyrosine hydroxylase (TH) immunoreactivity.

The intraperitoneal administration of the α7-nAChR-selective antagonist methyllycaconitine reversed these neuroprotective effects. This confirmed the implication of α7-nAChRs in curcumin-mediated effects. In this study it was shown that curcumin has a neuroprotective effect in a 6-hydroxydopmine (6-OHDA) rat model of PD via a α7-nAChR-mediated mechanism [90]. Zhang et al. have demonstrated that the expression of G2385R-LRRK2 induced neurodegeneration in human neuroblastoma SH-SY5Y and mouse primary neurons. This neurotoxicity mediated by oxidative stress results in the activation of the apoptotic pathway. Curcumin, which exhibits antioxidant activity, has significantly protected against the combined G2385R-LRRK2-induced neurodegeneration by attenuating the mitochondrial ROS levels, caspase-3/7 activation, and PARP cleavage and reducing the cellular environmental stressor H_2_O_2_ (Figure 2). These results provide new insight into the mechanisms of G2385R-LRRK2-related neurodegeneration and a potential therapeutic effect of curcumin in PD patients carrying G2385R [91].

In addition to the above-discussed curcumin-neuroprotective mechanisms against PD, a new growing interest in the gut-brain axis in PD could explain the neuroprotective properties of curcumin in spite of its limited bioavailability. Actually, curcumin can act indirectly on the CNS via the microbiota-gut-axis. The complex bidirectional system which playing an essential role in the brain health remains not fully understood.

Recent studies have shown that curcumin restores the dysbiosis of gut microbiome. Dysbiosis is defined as stable microbial community condition that functionally contributes to the etiology, diagnosis or treatment of a disease [92]. However, modifications of curcumin by bacterial do not form more active metabolite of curcumin [93]. This mutual interaction could maintain a balanced physiological functions and play a key role in neuroprotection and prevention against PD development and progression. Despite the increased, research interest in PD-associated non-motor symptoms such as depression, olfactory deficit, constipation, sleep, behavior disorder the effects of curcumin on PD needs further investigations.

Taken together, curcumin showed promising effects in the treatment of PD (Table S1) (see Figure 1). However, exploring more curcumin formulations in in vivo models and in clinical trials would provide further advancement in the use of curcumin as a preventive therapy to block or slow the onset of PD.

## 4. Curcumin as a Therapeutic Candidate in MS

MS is a chronic, neuroinflammatory, autoimmune demyelinating disease of the CNS in young adults that affects millions of people [94]. MS is associated with several pathophysiological processes including chronic inflammation, altered immune system, breaching of the BBB as relapsing-remitting (RR) episodes, infiltration of large number of leukocytes, oxidative stress, demyelination that consequently leads to axonal and neuronal damage, remyelination and repair systems activation [95,96,97,98]. Although the underlying cause of MS is still unknown, scientists believe that MS is a multifactorial disease that involves a combination of genetic, environmental, and autoimmunological factors that contribute to the risk of developing MS [99]. The initial phase of inflammation is characterized by the contribution of IL-22, IL-17 and T cells leading to the activation of an inflammatory cascade and other pathophysiological MS features, which are cause of the demyelination and axonal damage [100].

To date, only symptomatic treatment is available for MS, which focuses on treating relapses and remitting episodes of illness. Current MS treatment is known as disease-modifying therapy (DMT) in which various compounds have been developed. Most of these therapies are immunomodulatory compounds, approved for the treatment of different types of MS and target different pathophysiological pathways [101,102]. Other treatment strategies are being used involving the used stem cell therapy as autologous hematopoietic stem cell transplantation (aHSCT) and the B-cell depleting monoclonal therapies [102]. Relapses are the dominant clinical feature of RRMS, but also occur in the initial phase of secondary progressive MS [103]. The choice of treatment strategy for relapsing and remitting MS (RRMS), present in 85–90% of patients with MS, remains controversial [104]. This is due to the variability of the associated symptoms with MS for each individual. Despite the numerous therapies available, new challenges have been raised concerning the identification of the appropriate therapeutic strategy for each individual case. In addition, the safety and efficacy profile for these compounds, as well as the understanding of possible side effects remain challenging. The side effects, therapy failures, toxicity reports and the high cost of current chemical drugs are factors that favor the consideration of medicinal plants, including curcumin, for therapeutic purposes. Several properties of curcumin have recently been identified, some of which may be effective in treating MS, particularly its anti-inflammatory properties by inhibiting the secretion of pro-inflammatory cytokines (Figure 1) [103]. Here, we are going to review the various properties and main effects of curcumin for treating MS (Table S1). Given the indispensable role of astrocytes in the improvement and recovery from MS, the human astrocyte cell line (U373-MG) was used as the cellular model of MS in an earlier study [105]. In cells pretreated cells with LPS, curcumin reduced the release of both IL6 and MMP9 activity, although it did not affect either insulin-like growth factor (IGF)-1 nor neurotrophin-3 mRNA levels. This supports the anti-inflammatory effect of curcumin on astrocytes in the CNS [106]. The experimental autoimmune encephalomyelitis (EAE) produced by injection of myelin into mice was used as an experimental model to study MS. Interest in curcumin as a potential therapeutic candidate for MS is also growing. Interestingly, recent findings on the effects of curcumin on Lewis rat models of EAE have shown that polymerized nanoCUR (PNC) administered at dosage 12.5 mg/kg had an efficient therapeutic effect with significant effects on the EAE scores and showed myelin repair mechanisms. In fact, PNC increased myelination through an enhanced repair mechanism that induces enhanced neurotrophic factors. In addition, it reversed EAE-induced neuroinflammation by inhibiting the pro-inflammatory gene expression NF-ĸB, IL-1, IL-17, TNF-α, MCP-1 and increasing the anti-inflammatory gene expression IL-4, IL-10, FOXP3 and TGF-β. In addition, PNC modulated the expression of oxidative stress markers. More interestingly, pretreatment with PNC has increased the progenitor cell markers and delayed EAE development [27,107,108]. Given the importance of oligodendrocytes and their immature progenitors, which are important targets for therapeutic strategies for the treatment of demyelinating diseases, the effects of curcumin on oligodendrocytes were studied. Investigation of the effects of curcumin on the differentiation of oligodendrocyte progenitor (OP), particularly in inflammatory diseases, has shown that curcumin improves the differentiation of OPs through the increased expression of the markers associated with different developmental stages. Curcumin was able to activate PPAR-γ in OPs by showing a curcumin-dependent nuclear translocation of PPAR-γ [109]. The ability of curcumin to promote the differentiation of OPs into (immature oligodendrocytes) OLs involved several mechanisms, including PPAR-γ and ERK1/2 activation and prevention of TNF-α-induced deleterious effects. A recent study has confirmed the effectiveness of the nanoformulation of curcumin on the inflammatory characteristics in patients with MS. Indeed, curcumin significantly decreased the expression of miRNAs including miR-145, miR-132, miR-16, as well as inflammatory mediators such as: STAT-1, NF-kB, AP-1, IL-1β, IL-6, IFN-γ, CCL2, CCL5, TNF-α. On the other hand, nanoCUR has induced a significant increase in expression levels of Sox2, Sirtuin-1, Foxp3, PDCD1. In addition, the secretion levels of IFN-γ, CCL2, and CCL5 were drastically reduced in the patient group treated with curcumin compared to the placebo group [110]. T helper 1 (Th1) and T helper 17 (Th17) cells are involved in the MS pathogenesis and are believed to be therapeutic targets [111] (see Figure 2). Recent research on EAE models and MS patients highlighted a critical role for Th17 cells in mediating autoimmune neuroinflammation. Th17, the pro-inflammatory lineage of effector Th cells is believed to be the most important cytokines producer of IL17 [112]. Hence, these cells are involved in demyelination and axonal/neuronal degeneration. Interestingly, when compared to placebo group, the proportion of Th17 cells and the expression level of RORγt and IL-17 were significantly decreased in MS patients who received weekly interferon β-1a (Actovex) injections and supplemented with NanoCUR for 6 months [113]. Predominantly, EDSS score in the group of MS patient who were supplemented with nanoCUR showed a better quality compared to the placebo group. Overall, nanoCUR can inhibit disease progression in MS patients. In conclusion, nanoCUR could potentially be viewed as a neuroprotective agent against the progression of MS, primarily targeting the inflammatory properties of MS. Other studies using EAE models have suggested the central role of CD4^+^ regulatory T (Treg) cells in MS pathogenesis and exacerbation [114,115,116,117]. It is important to emphasize that the frequency and suppressive function of Treg cells are impaired in patients with MS [118,119]. Another recent study by Dolati et al. described nanoCUR effects on Treg function and frequency in patients with MS. A group of them received nanoCUR capsules effects for at least six months, another group received a placebo as a control group. An increased frequency of circulating Treg with higher expression of FoxP3 has been observed in MS patients. Overall, nano-formulation of curcumin was able to lower the EDSS score in MS patients compared to baseline, suggesting recovery from relapse events rather than real improvement. Based on the above results, it is found that nanoCUR is considered an immunomodulatory agent by regulating the function of immune system function and preventing the autoreactivity by modulating the proportion and function of Treg cells in MS patients [120]. These observations show that nanoCUR is able to of restore the frequency and function of Treg cells in MS patients, highlighting the emerging therapeutic mechanisms of curcumin in the MS treatment as strategy to promote remyelination.

## 5. The Therapeutic Effects of Curcumin in Glioblastoma Multiforme

Glioblastoma (GBM) is the most aggressive diffuse glioma of the astrocytic lineage and is classified as grade IV glioma according to the WHO classification [121]. GBM is the most common malignant primary brain tumor and accounts for 54% of all gliomas and 16% of all primary brain tumors [122]. GBM remains an incurable tumor with a survival rate of 14–15 months after diagnosis [123,124]. Despite advances in surgical resection, the prognosis for patients with GBM remains poor and dismal [125].

The standard approach for GBM treatment is maximal surgical resection followed by daily postoperative radiation and chemotherapy. Temozolomide, an oral alkylating agent that can cross the BBB, is the most common first-line treatment for GBM after surgery. It is used in combination with radiation therapy [126].

Given the invasive nature of the metastatic potential of GBM, complete resection of the tumor is difficult. Many factors can influence the effectiveness of these combined therapeutics, including the poor brain-targeted efficiency and multidrug resistance (MDR), which causes GBM cells to exhibit significantly poor monotherapy response even when relapsed from the resected marginal cavity [127]. In fact, the effectiveness of the chemotherapy drug temozolomide (TMZ) is often limited by drug resistance and the increasing adverse effects [128,129]. Therefore, GBM treatment remains challenging when there is an urgent need to improve chemotherapy outcomes and to identify new potential targets for GBM treatment.

Recent studies have shown that curcumin not only plays anti-cancer effects in lung, rectal and breast cancer, mainly due to its antioxidant and anti-inflammatory properties, but also because it increases the effectiveness of radiation and chemotherapy, leading to an improvement in survival as well as the expression of anti-metastatic proteins [130], and reducing, at the same time, their side effects [131,132,133,134]. Interestingly, curcumin enhances and triggers the apoptotic activity against the tumor cells involving the intrinsic and the extrinsic pathways as previously described [10,135]. Therefore, combination of curcumin with chemotherapy or radiotherapy could prime the sensitivity of cancer cells to chemotherapy or radiation therapy and improve the effectiveness of chemotherapy drugs.

Indeed, the expression of caspase-3 and Bax was increased, but the expression of Bcl-2, HIF1 in U251 cells was decreased after treatment with 20 and 30 μM curcumin. Both HIF-1α and ENO1 expression in U251 cells decreased. In hypoxic conditions, HIF-1αcan act as the main transcription factor activing the encoded glycolytic enzymes including ENO1.

It is well documented that increased glycolysis is considered to be one of the metabolic properties of GBM [136]. Enolase is an important glycolytic enzyme and ENO1 is its major isoform, which is expressed in GBM. In the same study, ENO1 was reduced resulting in the suppression of the growth, migration and invasive progression of glioma cells. In conclusion, ENO1 could be a potential target gene for curcumin and its anti-cancer mechanisms could be related to glycolytic and apoptotic pathways [137].

These findings were corroborated by recent research data showing that both nanomicelle-curcumin and curcumin in combination with Erlotinib reduce the viability, migration and invasion of human glioblastoma cells U87 in vitro. Both invasion and migration play an important role in cancer metastasis. Interestingly, the expression of angiogenesis-associated factors including VEGF, HIF-1 α, bFGF, and Cox-2 was markedly reduced in U87 human glioblastoma cells. On the other hand, curcumin alone or in combination with Erlotinib increased the expression of autophagy-associated proteins (LC3-II, LC3-I, and Beclin1) and modulated the expression of the pro-apoptotic factors Bax, Caspase 8, and Bcl-2 with the pro-inflammatory NF-κB (see Figure 2) [138].

In addition, the expression of the genes related to Wnt pathway such as cyclin D1, ZEB1, β-catenin, and Twist appeared significantly downregulated by curcumin [139].

At the molecular level, curcumin has been shown to suppress the proliferation of GBM cell proliferation via the AKT/mTOR signaling pathway and to increase PTEN expression. The in vitro experiments of this study have consistently confirmed that curcumin inhibits the migration and invasion of U251 cells derived from a human malignant Glioblastoma multiforme and stimulates apoptosis [140].

Various approaches have been proposed to achieve improved BBB penetration and effective intracephalic drug release, and to provide effective, targeted therapeutic agents for GBM. In these approaches, curcumin was encapsulated in surface-modified polyamidoamine (PAMAM) dendrimers of the fourth generation. Notably, in vitro use of encapsulated-curcumin at therapeutic doses has significantly reduced the viability of various glioblastoma cells from three different species (U98, F98 and GL261) [141]. It is known that cancer cells require a high oxidative state in order to maintain their growth and proliferation. As described above, curcumin is a nutraceutical compound known for its anti-inflammatory and antioxidant activities and therefore could be a new alternative potential candidate for the treatment of devastating GBM. However, the assessment of the potential of curcumin for GBM is linked to other existing treatments, but requires a future in vivo study with rodent models of glioblastoma.

To improve BBB penetration and to achieve efficient drug delivery to mouse glioblastoma, a rabies virus glycoprotein polypeptide derivative (RVG) peptide-directed, doxorubicin-loaded and curcumin-assisted reduction sensitivity nanomicelle (DOX/RVG-CSC) has been used. An appropriate delivery of curcumin stimulates the overall repolarization of microglia, which in turn stimulates the transformation of GBM cells from an immunosuppressive state M2 to a susceptible phenotype M1 [142]. Because of its unique microenvironmental compatibility and affinity for intracerebral gliomas, chondroitin sulfate (CHS) was used as the hydrophilic segment [143] and conjugated to curcumin via disulfide bonds. This led to spontaneously self-assembled core–shell polymeric micelles in water. RVG-mediated DOX/RVG-CSC penetrate the BBB, reach the target regions of tumor cell and then, after stimulation by high glutathione concentration in GBM, release the active drug [144].

In addition, recent findings show that curcumin can play an essential role in eliminating residual GMB cells by stimulating the immune system [145,146].

This emerging role of curcumin in the context of GBM was explored through a series of mechanistic studies performed in mouse models of GBM. More recently, Baidoo et al. have studied the use of the innate immune system in a therapeutic approach to the elimination of cancer cells. They discovered that tumors carry macrophages and microglia in their niches, but mostly in the state of tumor-promoting M2 under the control of tumor-released cytokines. The most notable finding emerging by their results is that curcumin induced the repolarization of tumor-associated macrophages (TAM) into the nitric oxide (NO)-producing tumoricidal M1 phenotype. This M2→M1 switch involved the curcumin-mediated suppression of STAT-3 and induction and activation of STAT-1. This recruits the activated natural killer cells (NK) and the cytotoxic T (Tc) into the tumor and consequently eliminates both the cancer cells and the cancer stem cells.

As such, this approach may provide a general strategy for combating GBM, but more studies are needed to better understand the implications of various related factors to curcumin-anticancer pathways [147,148,149,150].

Additionally, this has opened the prospect of a Phase I/II clinical trial in GBM patients to investigate the effectiveness of their curcumin-based therapy to induce repolarization of the TAMs.

In summary, curcumin is able to modulate GBM-associated pathways. For example, curcumin suppresses tumor growth by blocking tumor-promoting pathways NF-kB, PI3k/Akt/mammalian target of rapamycin (PI3K/Akt/mTOR), Janus kinase/signal transducers, and activators of transcription (JAK/STAT3) and mitogen-activated protein kinase pathways, while the major tumor-suppression genes (i.e., p53 and p21, and caspase) were up-regulated [151].

Consistent with all of the in vitro findings of curcumin, other beneficial in vivo effects of curcumin on GBM have been reported (Table S1), including the inhibition of matrix metalloproteinases (MMP)-dependent cell migration and invasive cell proliferation, which subsequently led to a reduced tumor volume and, at the same time, a longer survival time [137].

All discussed curcumin effects indicate that the functions/activities of glioblastoma cells are modulated, and their progression is delayed (Figure 1). However, genome profiling of glioblastoma tumors and the identification of specific targets of curcumin for GBM treatment remain important in understanding its pharmacological mechanisms and, more importantly, can provide a theoretical basis for the rational use of curcumin in clinical practice. Further research should be considered for a final conclusive report on the therapeutic effects of curcumin in clinical practice alone or in combination with medicinal products. The possible indirect effects on brain health and glioblastoma prevention through via the gut-brain axis require further investigation.

## 6. Curcumin and Epilepsy

Diseases of CNS are currently a major social and individual problem. In particular, the latest epidemiological evidence suggests that epilepsy constitutes an increasingly widespread group of diseases worldwide. For this reason, over the years, more and more drugs and therapies have been developed to counteract the symptoms and frequency of epileptic seizures; however, many of these drugs have been shown to be effective, but they are also responsible for serious and frequent side effects. In fact, many medicinal plants have been studied recently, and curcumin is one of these. Curcumin appears to play a role in the regulation of brain monoamine levels and this would suggest possible protective effects on seizure control and cognitive impairment (particularly with regard to memory disorders). Curcumin has been shown to have an antioxidant effect 10 times greater than vitamin E and represents a valid alternative to vitamin E itself [152].

Curcumin, is indeed able of inhibiting NF-kB mediated transcription, inflammatory cytokines, inducible iNOS and Cox-2 resulting in its antioxidant and anti-inflammatory properties [153]. These properties suggest its role in neuroprotection and neuromodulation in the epileptogenesis processes described (Table S1) (Figure 1).

The anti-epileptic action of curcumin could also be achieved through the upregulation of anti-inflammatory genes such as Interleukin-10 receptor subunit beta gene, chemokine ligand16 (CXCL16) and CXCL17 and NCSTN [154]. Recent preclinical studies have shown that curcumin may play a useful role in epilepsy and its associated disorders with no side effects or adverse effects [155,156]. Some experimental studies based on induced epilepsy model have reported the effectiveness of curcumin in delaying or completely inhibiting the onset of seizures [157].

Curcumin has also been suggested to play a role in determining the downregulation of some channel proteins (CACNA1A and GABRD), resulting in subsequent inhibition of FeCl_3_-induced seizures (Figure 2). Administration of curcumin reproduces human models of post-traumatic epilepsy [158].

Micronized curcumin has shown an efficacy comparable to that of the anti-epileptic drug valproate in inhibiting tonic-clonic seizures in PTZ-induced models of epilepsy in both larvae and adult zebrafish [159].

In another study, the evaluation of the anti-inflammatory and anticonvulsant effect of curcumin following high doses of FeCl_3_, administered with the diet and measured in parts per million (1500 ppm) showed superior efficacy in inhibiting generalized seizures compared to low doses (500 ppm) [160].

In a model of increasing-voltage electroshock test in mice, curcumin at a dose of 100 mg/kg orally increased the seizure threshold in both acute and chronic epilepsy (for 21 days) [161]. This effect is comparable to the administration of phenytoin (25 mg/kg PO) [161].

In this study, a reduction in mortality was found even with chronic curcumin administration, explain the anticonvulsant effect of this substance. Further pre-clinical studies confirmed its anticonvulsant and anti-inflammatory effect.

Furthermore, curcumin was found to play a protective role in reversing various oxidative stress changes associated with pilocarpine stimulation [162]. These data were also confirmed by another study in which evaluating curcumin doses between 100 and 300 mg/kg which were found to be useful in reducing pilocarpine-induced seizures [163].

Curcumin has also shown its effects in the epileptic state. Indeed, the study by Gupta et al. [164], predicted administration of curcumin in a dose range of 50–200 mg/kg approximately 30 min before stimulation with kainic acid. The authors of this study observed the protective effect of curcumin in increasing the latency of the onset of seizures when administered at doses between 100 and 200 mg/kg. The same group showed a statistically significant reduction in the incidence of seizures [164].

Lower doses showed no clinical effectiveness. Analysis of animal brains then showed how long-lasting seizures raised MDA levels and lowered glutathione levels. This effect could only be reversed with doses of 100 and 200 mg/kg of curcumin. Lower doses were not clinically useful [164].

Curcumin has also been shown to be effective in reducing cognitive decline and oxidative stress caused by the chronic use of antiepileptic drugs such as phenobarbital and carbamazepine which are widely used in clinical practice [165].

In addition, the efficacy of curcumin in epilepsy-associated disorders was also confirmed by studies with male Wistar rats in the PTZ-induced method. In this study, the administration of 300 mg/kg of curcumin resulted in both the improvement in the onset of PTZ-induced seizures and a reduction in oxidative stress and a decrease in the cognitive decline [165].

As known, the chronic administration of some antiepileptic drugs such as carbamazepine and phenobarbital, may cause a cognitive decline that was believed to be caused by oxidative stress. Curcumin, when administered together with these antiepileptic drugs has been shown to reverse this cognitive decline as well as oxidative stress parameters [165].

Other studies, examining the efficacy of curcumin in neurological and psychiatric disorders including cognitive decline, showed that no progression of cognitive decline was observed in mice who ingested curcumin compared to the cognitive decline in the mice who ingested phenytoin [166].

The injection of piperine together with curcumin could improve its bioavailability and make its anti-epileptic action even more effective [167].

These studies are very encouraging and represent the basis for future research despite there are limits both regarding the difficult reproducibility of human epileptogenic networks starting from experimental-based models and the difficulty in converting the doses administered in experimental models into doses for the humans.

## 7. Conclusions

The natural compound Curcumin has anti-oxidant, anti-inflammatory proprieties, and has protective effects by acting on various cellular pathways. In this review we focused our attention on the therapeutic effects of curcumin in neurodegenerative disorders such as AD, PD, MS, glioblastoma and epilepsy by modulating various molecular pathways in brain cells (see Table S1 and Figure 2). Extracellular vesicles or nanovesicles may improve the solubility and bioavailability of curcumin in the brain, but until now, the application of these new ways of curcumin delivery is not fully investigated in neurodegenerative diseases. Therefore, more research utilizing these therapeutic biomolecules may lead to give a positive outcome for neuroprotection. These new studies may be focalized on (1) improving drug delivery systems to enhance the bioavailability and BBB permeability of curcumin; (2) furthering clinical studies to establish the more effective dose of these biomolecules transporting curcumin for the treatment of neurodegenerative disorders; (3) investigating the signaling pathways that the therapeutic biomolecules utilize to induce neuroprotection. The results described in this review are encouraging but further research is needed to optimize the use of curcumin in the prevention and treatment of neurodegenerative diseases.

## Data Availability

The data presented in this study are available on request from the corresponding author.

## Supplementary Materials

The following are available online, Table S1: Summary of the major effects of curcumin in brain disorders and the main targeted signaling pathways.
