# Supplementary material for: New Promising Therapeutic Avenues of Curcumin in Brain Diseases
Source: Molecules. 2021 Dec 31;27(1):236. doi: 10.3390/molecules27010236 (PMC8746812; doi:10.3390/molecules27010236)
Supplement: Supplementary file 1 [file molecules-27-00236-s001.zip › molecules-1508488-supplementary.pdf]

Table S1. Summary of the major effects of curcumin in brain disorders and the main targeted signaling pathways.

Brain Diseases	Biological Activity	Signaling Pathways	References
Alzheimer's Disease	Reduction of Aβ production	Inhibition of BACE1	[28]
Alzheimer's Disease	Reduction of Aβ production	Inhibition of GSK-3β and PS1	[49]
Alzheimer's Disease	Reduction of Aβ production	Inhibition of PTEN mRNA overexpression, downregulation of activation of pAKT	[51,52]
Alzheimer's Disease	Reduction of Aβ neurotoxicity	Decreasing activation of NMDA receptor of glutamate	[51]
Alzheimer's Disease	Reduction of Aβ neurotoxicity induced by microglia activation	Blocking ERK1/2, p38 kinase, PI3K/AKT phosphorylation and NF-κB activation	[63,64]
Alzheimer's Disease	Regulation of neurogenesis	Increasing Wnt level, enhancing TCF/LEF and cyclin-D1 promoter activity	[68]
Parkinson's Disease	Reduction of α -synuclein level	Increasing the expression of LC3-II; TFEB, ALAMP2A	[83,84]
Multiple Sclerosis	Reduction of inflammation	Decreasing of pro-inflammatory gene expression (IL-1; IL-17; TNF-α: MCP-1); increasing of anti-inflammatory gene expression (STAT-1, NF-kB, AP-1, IL-1β, IL-6, IFN-γ, CCL2, CCL5, TNF-α)	[27,107,108]
Multiple Sclerosis	Reduction of inflammation	Decreasing of pro-inflammatory miRNAs (miR-145; miR132; miR-16); increasing of anti-inflammatory mediators (STAT-1, NF-kB, AP-1, IL-1β, IL-6, IFN-γ, CCL2, CCL5, TNF-α)	[110]
Multiple Sclerosis	Differentiation of oligodendrocyte progenitors	Activation of PPAR-γ and ERK1/2	[109]
Glioblastoma Multiforme	Enhancing apoptotic activity of tumor cells	Increasing the expression of caspase-3, Bax and decreasing the expression of Bcl-2, HIF1	[138]
Glioblastoma Multiforme	Suppression of the growth, migration, and invasive progression of glioma cells	Inhibition of PI3K/Akt pathway	[129]
Glioblastoma Multiforme	Reduction of angiogenesis	Reduction of expression of VEGF, HIF-1 α, bFGF, and Cox-2.	[139]
Glioblastoma Multiforme	Enhancing autophagy	Increasing the expression of LC3-II, LC3-I, Beclin1 and decreasing Wnt pathway (cyclin D1, ZEB1, β-catenin, and Twist)	[139]
Glioblastoma Multiforme	Suppression cell proliferation	Activation of AKT/mTOR signaling pathway and increasing PTEN expression	[140]
Glioblastoma Multiforme	Induction of the repolarization of tumor-associated macrophages (TAM), M2→M1 switch	Suppression of STAT-3 and induction of STAT-1	[149]
Glioblastoma Multiforme	Suppression of tumor growth	Blocking of NF-kB, PI3K/Akt/mTOR, JAK/STAT3 MAPK pathways	[151]
Glioblastoma Multiforme	Reduction of tumor volume	Inhibition of MMP	[137]
Epilepsy	Anti-inflammatory	Inhibition of NF-kB	[154]
Epilepsy	Anti-inflammatory	Upregulation of IL10RB, CXCL16, CXCL17 and NCSTN.	[155]
Epilepsy	Anti-inflammatory and anticonvulsant	Downregulation of some channel proteins CACNA1A and GABRD.	[159]
